# Supplementary material for: A Nodal enhanced micropeptide NEMEP regulates glucose uptake during mesendoderm differentiation of embryonic stem cells
Source: Nat Commun. 2022 Jul 9;13:3984. doi: 10.1038/s41467-022-31762-x (PMC9271079; doi:10.1038/s41467-022-31762-x)
Supplement: Supplementary file 1 — Supplementary Information [file 41467_2022_31762_MOESM1_ESM.pdf]

## Supplementary Information

Supplementary Information includes nine figures with legends, six tables and one supplementary data (Supplementary Data 1).

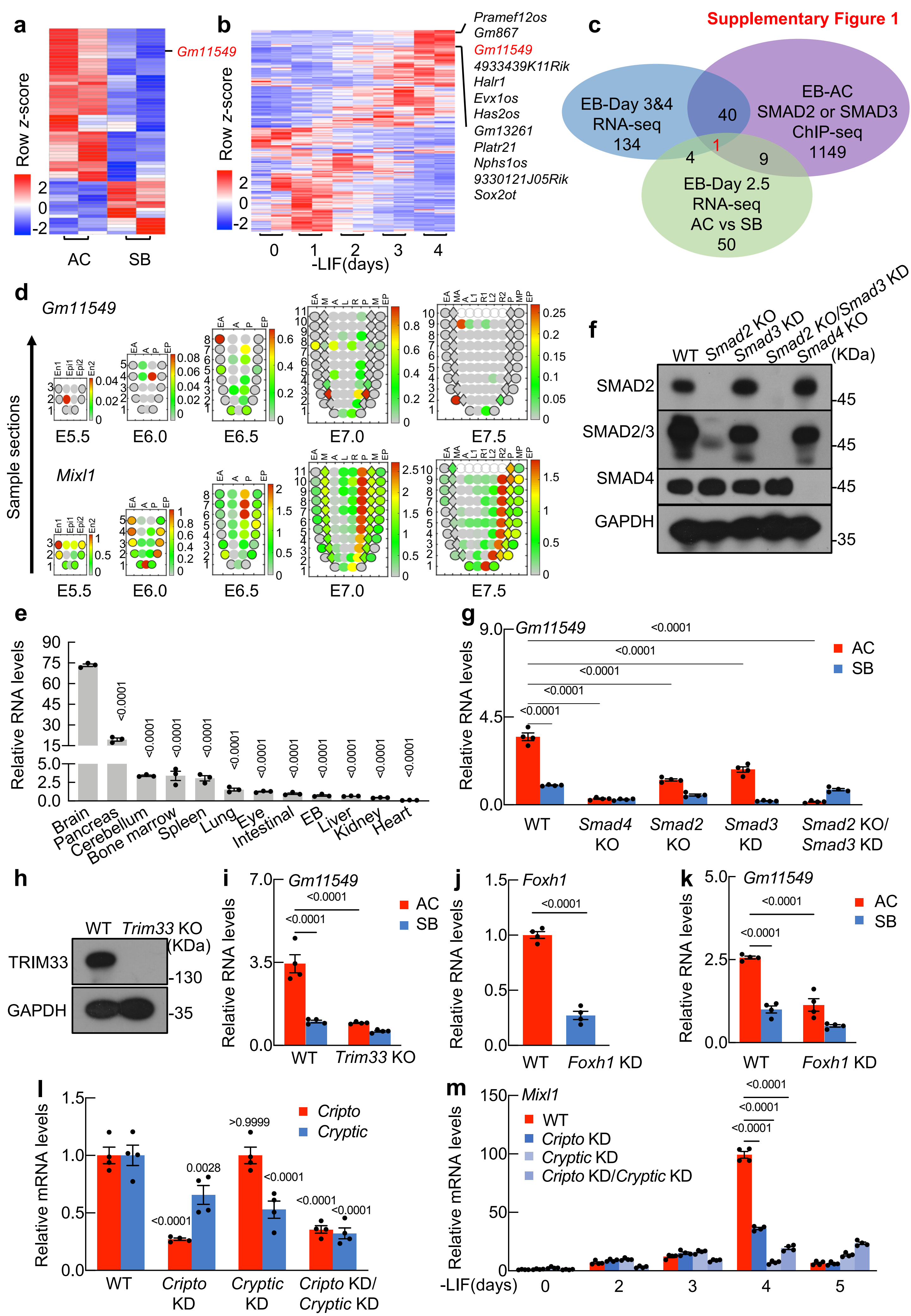
Supplementary Figure 1
a
Gm11549
Row z-score
AC
SB
b
Row z-score
-LIF(days)
Pramef12os
Gm867
Gm11549
4933439K11Rik
Halr1
Evx1os
Has2os
Gm13261
Platr21
Nphs1os
9330121J05Rik
Sox2ot
c
EB-Day 3&4 RNA-seq 134
EB-AC SMAD2 or SMAD3 ChIP-seq 1149
EB-Day 2.5 RNA-seq AC vs SB 50
40
1
4
9
d
Gm11549
Mixl1
Sample sections
E5.5
E6.0
E6.5
E7.0
E7.5
f
WT
Smad2 KO
Smad3 KD
Smad2 KO/Smad3 KD
Smad4 KO
(KDa)
SMAD2
SMAD2/3
SMAD4
GAPDH
-45
-45
-45
-35
e
Relative RNA levels
Brain
Pancreas
Cerebellum
Bone marrow
Spleen
Lung
Eye
Intestinal
EB
Liver
Kidney
Heart
<0.0001
g
Gm11549
Relative RNA levels
AC
SB
WT
Smad4 KO
Smad2 KO
Smad3 KD
Smad2 KO/ Smad3 KD
<0.0001
h
WT
Trim33 KO
(KDa)
TRIM33
GAPDH
-130
-35
i
Gm11549
Relative RNA levels
AC
SB
WT
Trim33 KO
<0.0001
j
Foxh1
Relative RNA levels
WT
Foxh1 KD
<0.0001
k
Gm11549
Relative RNA levels
AC
SB
WT
Foxh1 KD
<0.0001
l
Relative mRNA levels
Cripto
Cryptic
WT
Cripto KD
Cryptic KD
Cripto KD/ Cryptic KD
<0.0001
0.0028
>0.9999
m
Mixl1
Relative mRNA levels
WT
Cripto KD
Cryptic KD
Cripto KD/Cryptic KD
-LIF(days)
<0.0001

**Supplementary Fig. 1 related to Fig. 1: *Gm11549* is a direct target gene of Nodal signaling in mesendoderm differentiation of mouse ESCs.**

**a** The heatmap presentation of RNA-seq transcriptomic analysis of day 2.5 EBs with 2 h Activin A (AC) - and SB431532 (SB) - treatment for putative annotated lncRNAs (fold change (AC/SB) > 1.5 or < 0.5). *Gm11549* is indicated (n = 2 biological independent samples).

**b** The heatmap presentation of RNA-seq transcriptomic analysis of EBs from day 0 to day 4 for putative annotated lncRNAs (GEO: GSE70486) (n = 2 biological independent samples).

**c** Venn diagram showing numbers of overlapping and non-overlapping lncRNAs that are specifically expressed in day 3 & 4 EBs, have SMAD2 and SMAD3 binding at the promoter region and differentially expressed in day 3 EBs with 2 h Activin A (AC) - and SB431532(SB) - treatment with fold change (AC/SB) > 1.5 or < 0.5, $p < 0.05$ (n = 2 biological independent samples).

**d** Adapted from (Peng et al., 2019b): corn plots showing the spatial pattern of *Gm11549* and *Mixl1* expression at E5.5 to E7.5 mouse embryo. A, anterior; P, posterior; L, left lateral; R, right lateral; L1, anterior left lateral; R1, anterior right lateral; L2, posterior left lateral; R2, posterior right lateral; Epi1 and Epi2, divided epiblast; M, whole mesoderm; MA, anterior mesoderm; MP, posterior mesoderm; En1 and En2, divided endoderm; EA, anterior endoderm; EP, posterior endoderm.

**e** qPCR analysis of *Gm11549* expression in the indicated adult tissues of mouse (n = 3 biological independent samples).

**f** WT, *Smad2* KO, Smad3 KD, *Smad2* KO/*Smad3* KD, and *Smad4* KO mESCs were verified by immunoblotting analysis using antibody against SMAD4, SMAD2, SMAD2/3, and GAPDH.

**g** qPCR analysis of *Gm11549* expression in Activin A (AC) - or SB431542 (SB) - treated day 2.5 EBs derived from WT or *Smad2* KO, *Smad3* KD, *Smad2* KO/*Smad3* KD and *Smad4* KO cells for 2 h (n = 4 biological independent samples).

**h** *Trim33* KO mESCs were verified by immunoblotting analysis using antibody against TRIM33 and GAPDH. Date is representative of three independent experiments.

**i** qPCR analysis of *Gm11549* expression in Activin A (AC) - or SB431542 (SB) - treated day 2.5 EBs derived from WT or *Trim33* KO cells for 2 h (n = 4 biological independent samples).

**j** Validating *Foxh1* expression in WT and *Foxh1* KD mESCs by qPCR analysis (n = 4 biological independent samples).

**k** qPCR analysis of *Gm11549* expression in Activin A (AC) - or SB431542 (SB) - treated day 2.5 EBs derived from WT or *Foxh1* KD cells for 2 h (n = 4 biological independent samples).

**l** Validating *Cripto* and *Cryptic* expression in WT, *Cripto* KD, *Cryptic* KD, *Cripto* KD/*Cryptic* KD mESCs by qPCR analysis (n = 4 biological independent samples).

**m** qPCR analysis of *Mixl1* expression in day 0 to day 5 EBs from in WT, *Cripto* KD, *Cryptic* KD, *Cripto* KD/*Cryptic* KD cells (n = 4 biological independent samples).

Supplementary Figure 1 **e, f, g, i-m** : Data are the mean ± S.E.M. *P* values were determined by unpaired two-tailed *t*-test (**j**), one-way ANOVA (**e**) with Dunnett's corrections or two-way ANOVA with Sidak's corrections (**g**,**i**,**k**) or with Tukey's corrections (**m**) , and data are representative of three independent experiments with similar results.
Source data are provided as a Source Data file.

a

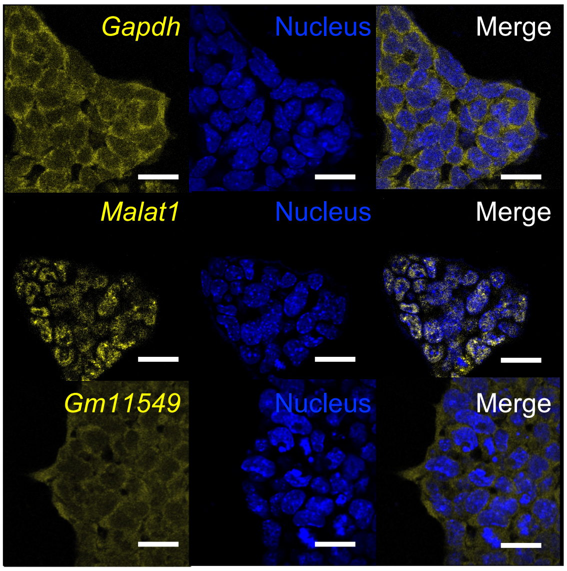


b

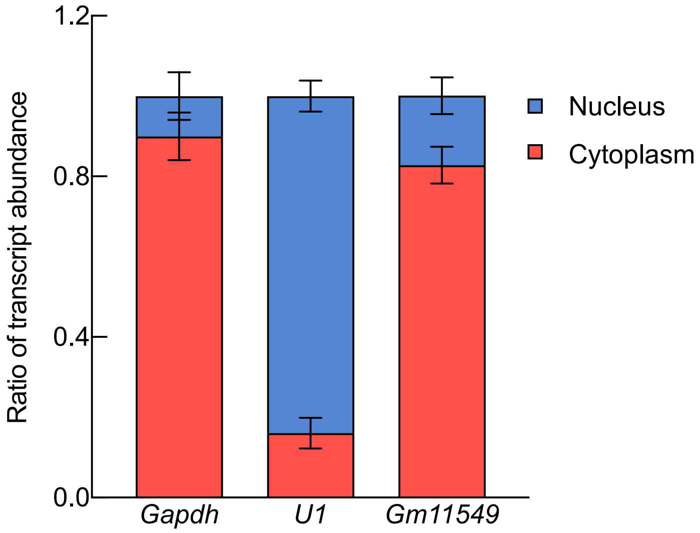


**Supplementary Fig. 2 related to Fig. 2: *Gm11549* transcript is enriched in cytoplasm**
**a** Subcellular localization of *Gm11549* detected by RNA FISH. *Gapdh* and *Malat1* transcripts mark the cytoplasmic, nuclear fractions respectively. Scale bar, 20 μm. The data is the representative of three independent experiments.
**b** Subcellular localization of *Gm11549* detected by fractionation assay. RNA was extracted from fractions (nucleus and cytoplasm) of differentiated ESCs at day 3. Subcellular distribution of *Gm11549* and the indicated marker transcripts. All RNA abundance values are absolute quantification. *Gapdh* and *U1* transcripts are the markers for cytoplasmic and nuclear fractions respectively. Data are the mean ± S.E.M of three biological independent samples, data are representative of three independent experiments with similar results.

## Supplementary Figure 3

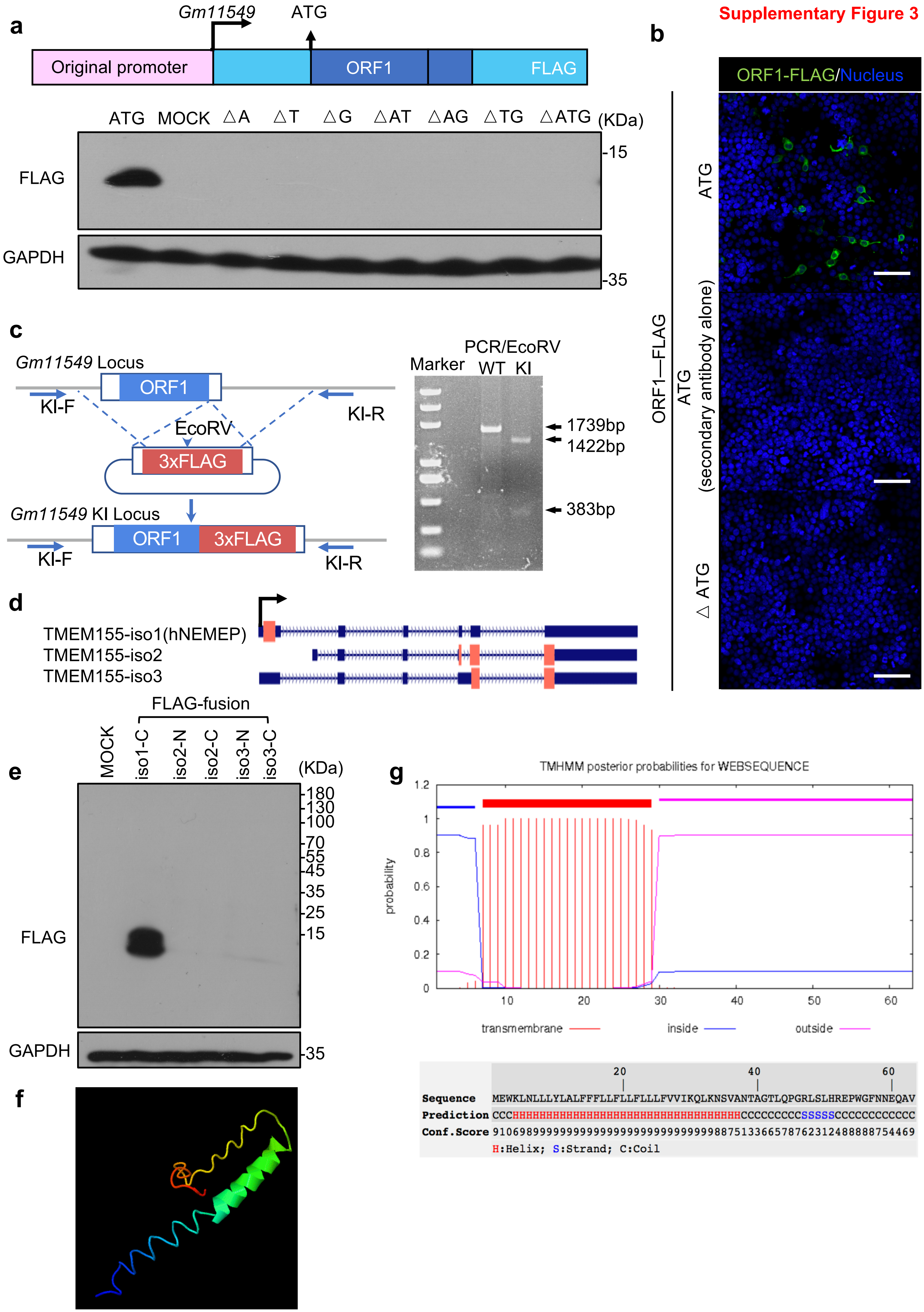

**Supplementary Fig. 3 related to Fig. 2: *Gm11549* encodes a transmembrane micropeptide: NEMEP.**

**a** Diagram of the ORF1-FLAG fusion constructs. The start codon ATG of the ORF1(ATG) was mutated to different variants (△A,△T,△G,△AG,△AT,△TG, or △ATG). The indicated constructs were transient expressed in HEK293T cells, and variant ORF1-FLAG fusion protein levels were determined by immunoblotting analysis using antibody against FLAG and GAPDH.

**b** Immunofluorescence analysis of transient expressed ORF1-FLAG and mutant variant in HEK293T. The staining of secondary antibody alone served as negative control. Scale bar, 100 μm.

**c** Left, scheme of generation of ORF1-3xFLAG knock-in mESCs strategy. The 3xFLAG was inserted after the last coding codon (GTG-valine) of ORF1 by CRISPR/Cas9-mediated homologous recombination in mESCs. Right, validating ORF1-3xFLAG knock-in day3 EBs by PCR.

**d** Predicted ORFs in the *TMEM155* RNA sequence.

**e** HEK293T cells were transiently transfected with expression vectors for all three of the predicted ORFs, each tagged with a FLAG epitope at their C termini. Immunoblotting analysis for accumulation of proteinaceous gene products from the predicted ORFs, detected with an anti-FLAG antibody.

**f, g** NEMEP is predicted to be a single-pass transmembrane protein using the TMHMM Server v.2.0 and I-TASSER.

Supplementary Figure 3**a**-**c**,**e** : Data are representative of three independent experiments with similar results.

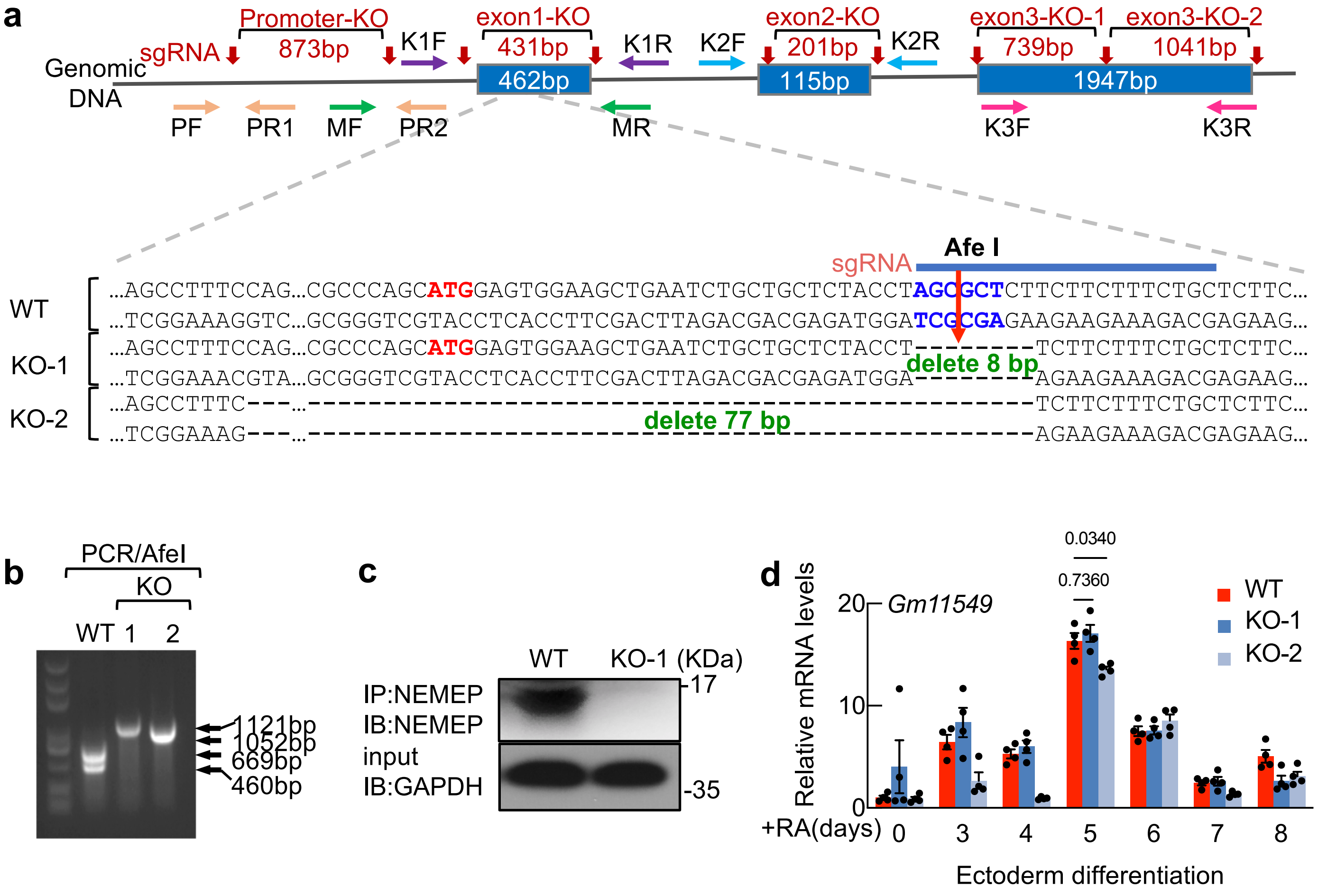


**Supplementary Fig. 4 related to Fig. 3: Genotyping of variant mutants of NEMEP mESCs generated by CRISPR-Cas9.**

**a** Scheme of gene locus of *Gm11549* and genome editing sites for variant mutants (gRNAs are indicated for indicated mutants in RED). DNA sequence alignments between WT and two frameshift mutants (KO-1 and KO-2) are showed.

**b** PCR analysis wild-type and CRISPR-Cas9 editing KO mESCs. Primers used are indicated in Supplementary Fig 4**a** (NEMEP frameshift mutants, promoter KO mutants, exon1 deletion mutant, exon2 deletion mutant, exon3 deletion mutant-1, and exon3 deletion mutant-2). The data is the representative of three independent experiments with similar results.

**c** Endogenous NEMEP was pull-down using a NEMEP polyclonal antibody raised against a 25-residue region of the NEMEP in WT and *Nemep* KO EBs at day 3. And Immunoblotting analysis of NEMEP revealed NEMEP expression in both cell lines. GAPDH was used as an internal control. The data is the representative of three independent experiments with similar results.

**d** *Nemep* KO and WT mESCs were induced for ectoderm differentiation for the indicated durations. Total RNA was analyzed by qPCR using primers for *Gm11549*. n = 4 biological independent samples. Data represents the mean ± S.E.M. *P* values were determined by two-way ANOVA with Dunnett's corrections. Data is representative of three independent experiments with similar results.

Source data are provided as a Source Data file.

Supplementary Figure 5

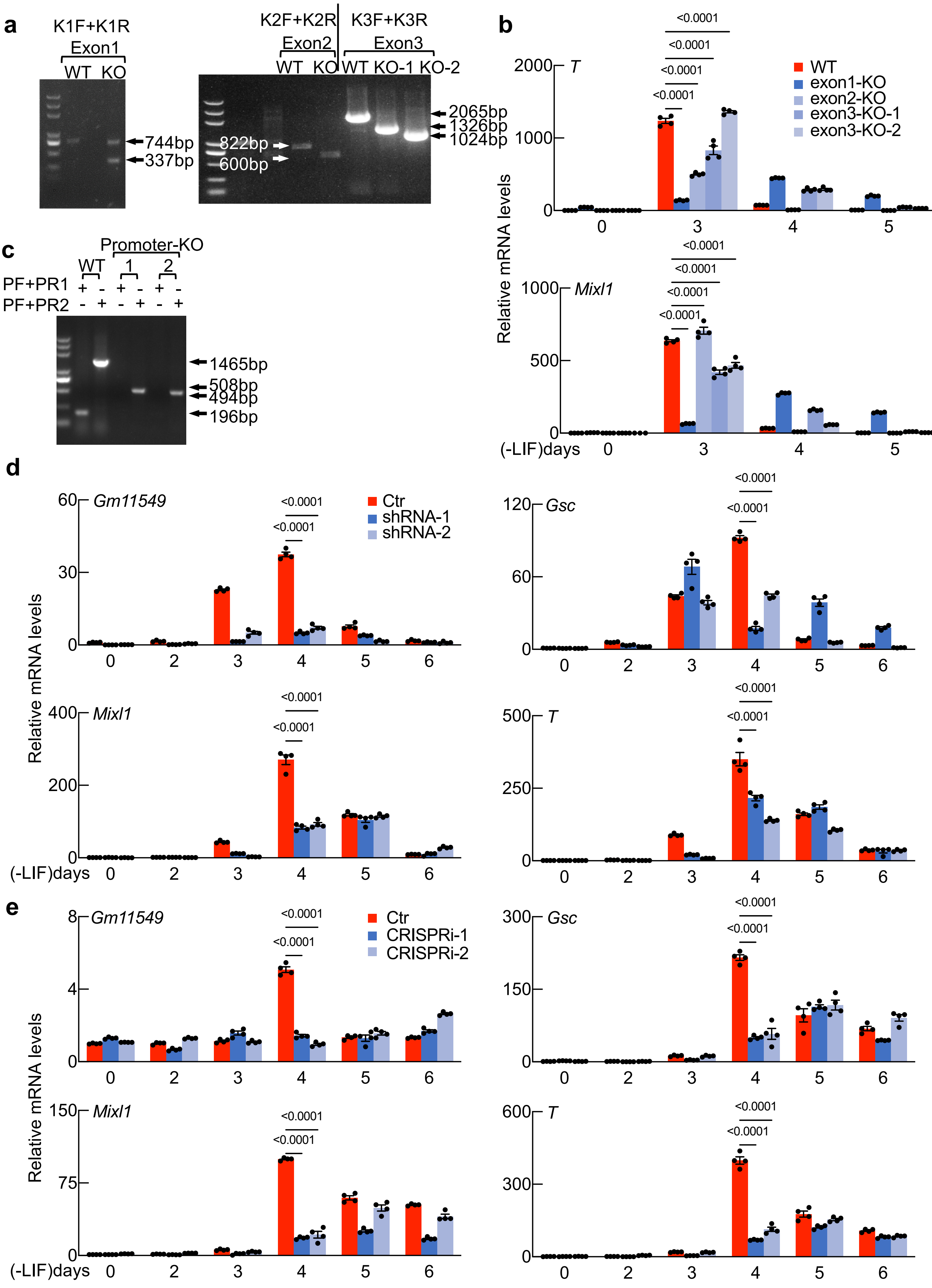

**Supplementary Fig. 5 related to Fig. 3: NEMEP is essential for mesendoderm differentiation.**

**a** PCR analysis wild-type and CRISPR-Cas9 editing exon1 deletion mutant, exon2 deletion mutant, exon3 deletion mutant-1, and exon3 deletion mutant-2 mESCs. Primers used are indicated in Supplementary Fig. 4**a** (NEMEP frameshift mutants, promoter KO mutants, exon1 deletion mutant, exon2 deletion mutant, exon3 deletion mutant-1, and exon3 deletion mutant-2).

**b** WT and various truncation variants of *Gm11549* mESCs (*i.e.*, variants lacking for exon1, exon2, and exon 3 sequences, etc.) generated by CRISPR/Cas9 system (Supplementary Fig. 3**a**, **e**) were induced for EB formation for the indicated durations. Total RNA was analyzed by qPCR using primers for the indicated genes.

**c** PCR analysis wild-type and CRISPR-Cas9 editing promoter KO mutants mESCs. Primers used are indicated in Supplementary Fig. 4**a**.

**d** qPCR analysis of indicated genes expression in day 0 to day 6 EBs from WT or *Gm11549* KD cells by shRNA.

**e** qPCR analysis of indicated genes expression in day 0 to day 6 EBs from WT or *Gm11549* KD cells by CRISPRi.

Supplementary Fig. 5 **a**-**e**: Data are representative of three independent experiments with similar results. Supplementary Fig. 5 **b, d** and **e**: Data are the mean ± S.E.M of four biological independent samples. *P* values were determined by two-way ANOVA with Dunnett's corrections.

Source data are provided as a Source Data file.

Supplementary Figure 6

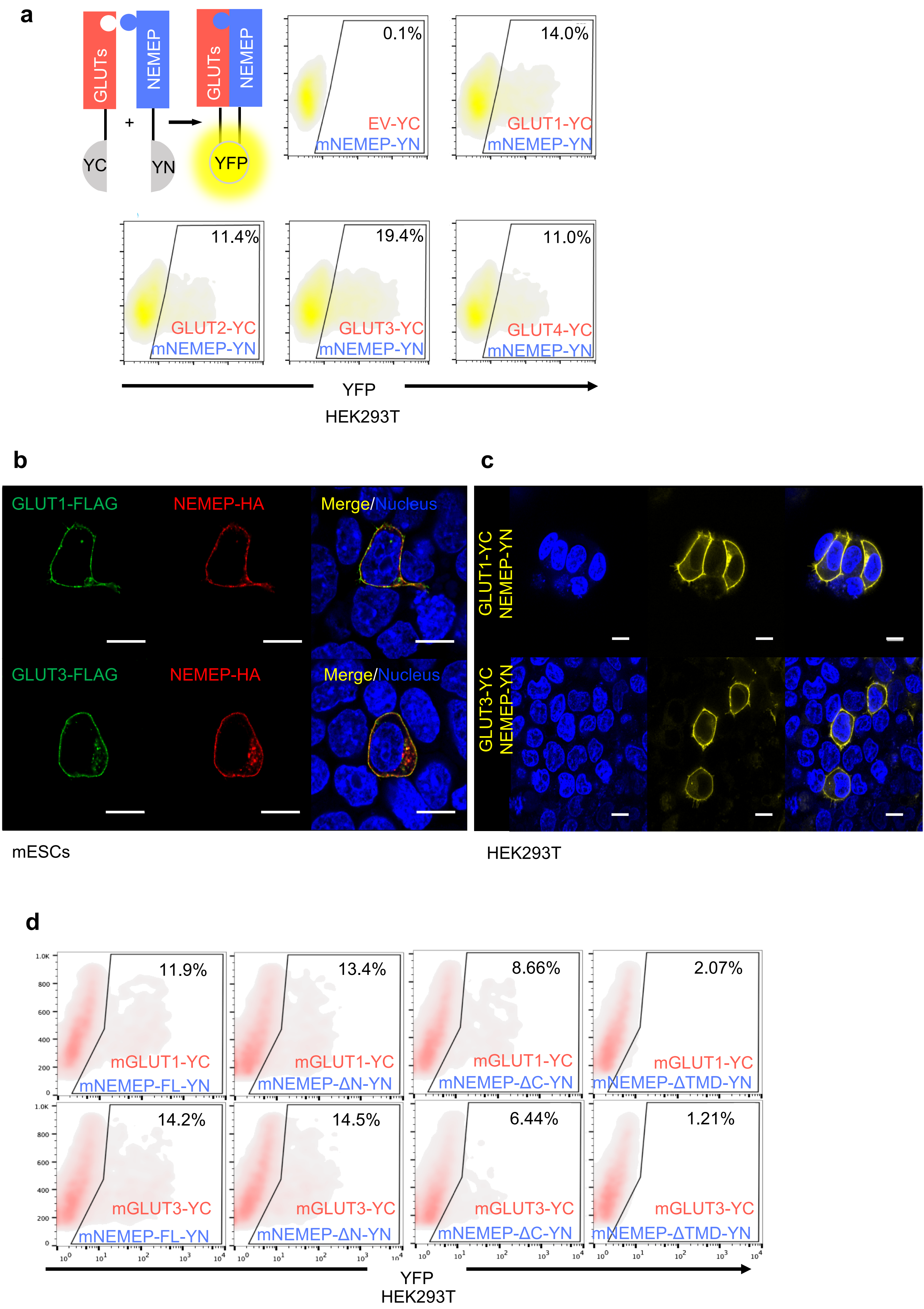

**Supplementary Fig. 6 related to Fig. 5: NEMEP interacts with GLUT1 and GLUT3 and colocalizes with GLUT1 and GLUT3 on the cell membrane.**

**a** Fluorescence-activated cell sorting (FACS) analysis of GLUTs (1-4)-NEMEP BiFC cells.

**b** Immunofluorescence detection of NEMEP-HA and GLUT1 and GLUT3-FLAG in mESCs transiently co-transfected with NEMEP-HA and GLUT1 and GLUT 3-FLAG vectors with anti-HA antibody (green) and anti-FLAG antibody (red). Nuclei are stained with Hoechst33342 (blue). Original magnifications, 10 μm.

**c**, **d** Fluorescence-activated cell sorting (FACS) and confocal microscope images analysis of GLUT1 and GLUT3-NEMEP BiFC cells. mGLUT1 and mGLUT3 are fused with YC, WT or mutants of mNEMEP are fused with YN. Scale bars, 10 μm.

Supplementary Fig. **6 a**-**d:** Data are representative of three independent experiments with similar results.

Source data are provided as a Source Data file.

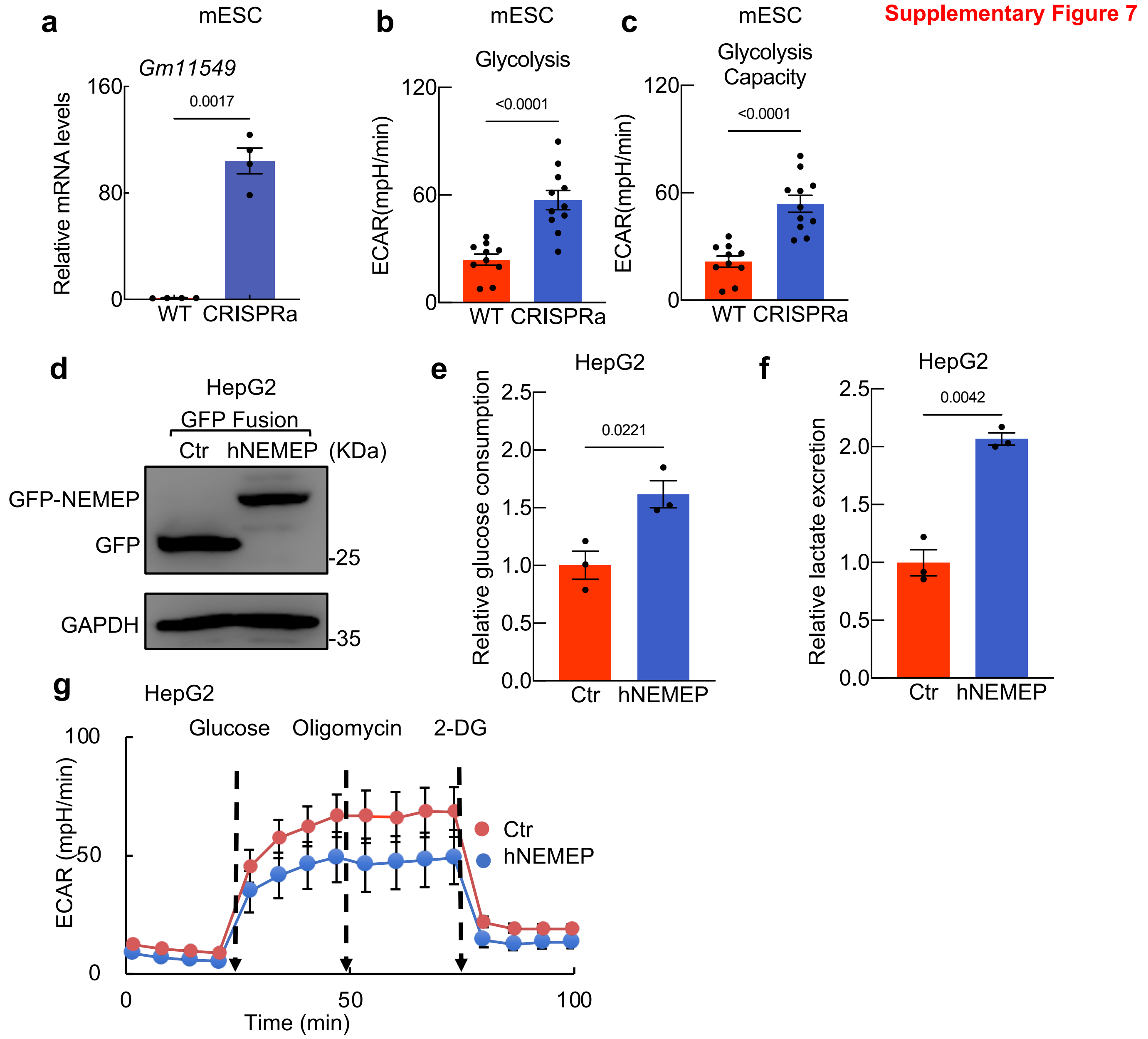


**Supplementary Fig. 7 related to Fig. 5: NEMEP facilitates glucose uptake.**

**a** Validating *Gm11549* expression in WT and *CRISPRa* mediated *Gm11549* overexpressing mESCs by qPCR analysis *(means* ± S.E.M.*,* n = 4 biological independent samples).

**b**, **c** WT and CRISPR-dCas9-VP64 (CRISPRa) mediated NEMEP overexpressing mESCs were supplied with 25 mM glucose*,* 2 μM of oligomycin (ATP synthase inhibitor), and 50 mM 2-DG (a glucose analog that inhibit glycolysis) for ECAR test. Relative glycolysis levels (**b**) and glycolytic capacity (**c**); these values are normalized to the protein concentration (means ± S.E.M.*,* n = 10 biological independent samples for WT, n = 11 biological independent samples for CRISPRa).

**d** Western blotting analysis of GFP and hNEMEP-GFP in control and hNEMEP-GFP overexpressing HepG2 cells using antibody against GFP. Data are representative of three independent experiments with similar results.

**e**, **f** Glucose consumption (**e**) and lactate excretion (**f**) in control and hNEMEP overexpressing HepG2 cells. *(means* ± S.E.M.*,* n = 3 biological independent samples)

**g** Control and hNEMEP overexpressing HepG2 cells were supplied with 25 mM glucose, 2 μM oligomycin, and 50 mM 2-DG at the indicted time. ECAR was examined using Seahorse XFe96 analyzer. Normalized to the protein concentration (means ± S.E.M., n = 8 independent samples).

Supplementary Fig. 7 **b**, **c**, **e-g**: Data are representative of three independent experiments with similar results. *P* values were determined by unpaired two-tailed *t*-test. Source data are provided as a Source Data file.

**Supplementary Figure 8**

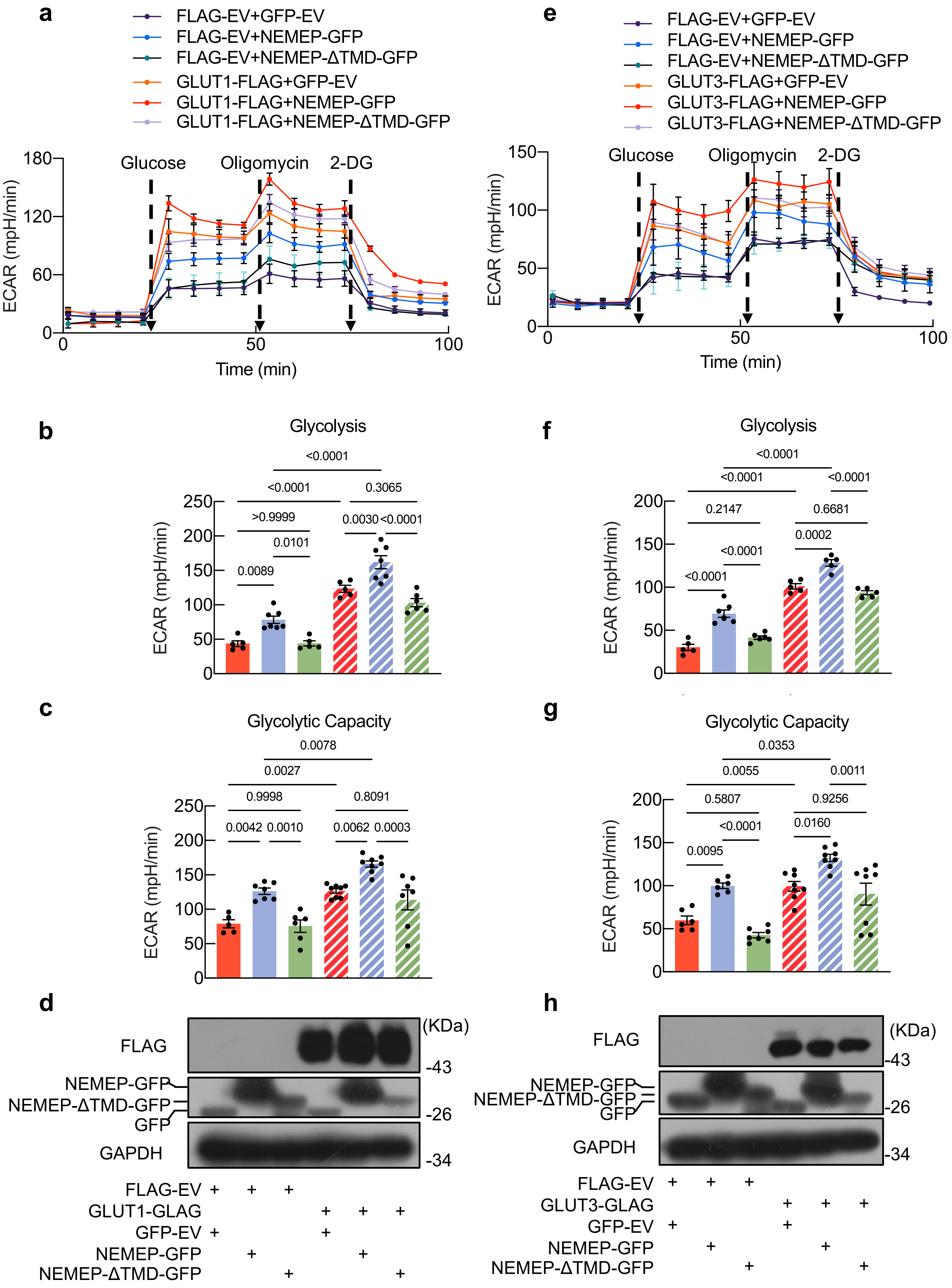

**Supplementary Fig. 8 related to Fig. 5: NEMEP interacts with GLUT1/ GLUT3 and facilitates glucose uptake.**

**a**-**c**, **e**-**g** HEK293T cells expressing plasmids for overexpression of the indicated proteins or protein pairs were supplied with 25 mM glucose, 2 μM oligomycin, and 50 mM 2-DG at the indicated time. ECAR was examined using Seahorse XFe96 analyzer (**a** and **e**). Relative glycolysis levels (**b** and **f**) and glycolytic capacity (**c** and **g**) are normalized to the protein concentration (means ± S.E.M., at least five biological independent samples per condition). *P* values were determined by two-way ANOVA with Tukey's corrections. Data are representative of three independent experiments with similar results.

**d** and **h** Lysates from HEK293T cells expressing plasmids for overexpression of the indicated proteins or protein pairs were analyzed by immunoblotting using antibodies against FLAG, GFP and GAPDH. Data are representative of three independent experiments with similar results.

Source data are provided as a Source Data file.

**Supplementary Figure 9**

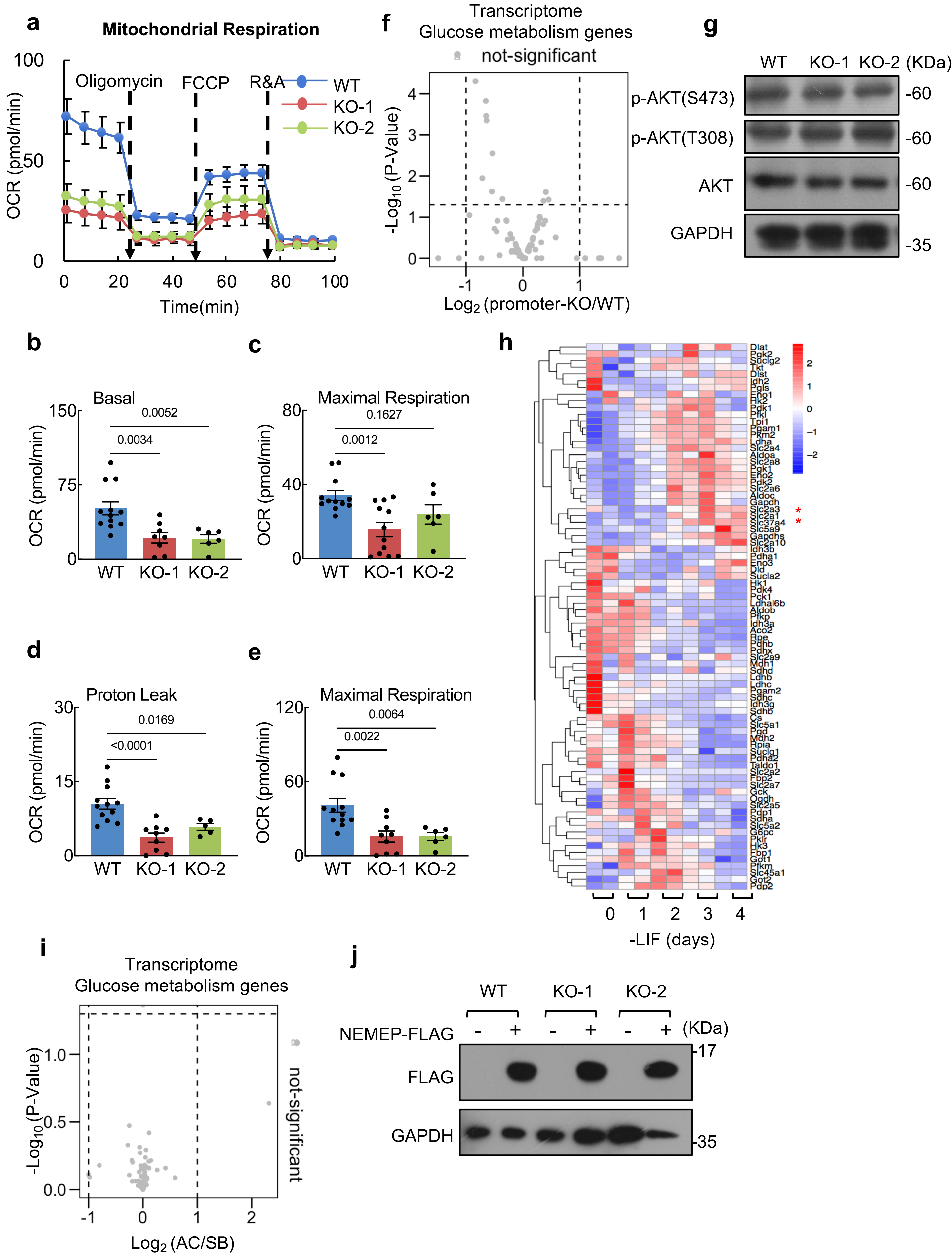

**Supplementary Fig. 9 related to Fig. 6 and 7: NEMEP facilitates glucose uptake during mesendoderm differentiation.**

**a**-**e** WT and *Nemep* KO EBs were supplied with 2 μM oligomycin, 2 μM FCCP (mitochondrial oxidative phosphorylation uncoupler), and 1 μM Antimycin A & 1 μM Rotenone (mitochondrial respiratory chain inhibitors) at the indicated time. OCR was examined using Seahorse XFe96 analyzer (**a**). Relative basal levels (**b**), maximal respiration (**c**), proton leak (**d**), and ATP production (**e**) are normalized to the protein concentration (means ± S.E.M., at least five biological independent samples per genotype). *P* values were determined by one-way ANOVA with Dunnett's corrections. Data is representative of three independent experiments with similar results.

**f** Volcano plot of glucose metabolism genes of day 3 EBs of WT and *Gm11549* promoter KO cells (n = 2 biological independent samples). Gray, transcripts non-significant changes (log2 |fold change| < 1, $p > 0.05$).

**g** Immunoblotting analysis of indicated proteins in WT and *Nemep* KO day 3 EBs. GAPDH was used as internal control. Data is representative of three independent experiments with similar results.

**h** The heatmap presentation of RNA-seq transcriptomic analysis of EBs from day 0 to day 4 for glucose metabolism related genes (GEO: GSE70486).

**i** Volcano plot of glucose metabolism genes of day 2.5 EBs with Activin A (AC) - and SB431542 (SB) - treatment (n = 2 biological independent samples). Gray, transcripts non-significant changes (log2 |fold change| < 1, $p > 0.05$).

**j** Lysates from NEMEP-FLAG overexpressing in WT and *Nemep* KO mESCs were analyzed by Immunoblotting using anti-FLAG antibody. GAPDH was used as internal control. Data is representative of three independent experiments with similar results.

Source data are provided as a Source Data file.

**Supplementary Table 1: RPKM of GLUT1 to GLUT4 in day 3 EBs (GSE157073).**

| | RPKM | |
|---|---|---|
| Gene | EBs day 3 - 1 | EBs day 3 - 2 |
| *Glut1* (*Slc2a1*) | 482.322 | 355.795 |
| *Glut2* (*Slc2a2*) | 0 | 0.00832 |
| *Glut3* (*Slc2a3*) | 319.229 | 219.159 |
| *Glut4* (*Slc2a4*) | 1.58826 | 1.58426 |

**Supplementary Table 2: shRNA oligonucleotides used in this study.**

| Target | NO. | Sequence (5'→3') |
|---|---|---|
| *Gm11549* | 1# | CCGGAAGTGTTGATTCACTACATCTCTCGAGAGATGTAGTGAATCAACACTTTTTTTG |
| | | AATTCAAAAAAAGTGTTGATTCACTACATCTCTCGAGAGATGTAGTGAATCAACACTT |
| | 2# | CCGGAATATTGTGAATCCCAAGCAATTCTCGAGAATTGCTTGGGATTCACAATATTTTTTTG |
| | | AATTCAAAAAAATATTGTGAATCCCAAGCAATTCTCGAGAATTGCTTGGGATTCACAATATT |
| *Glut1* (*Slc2a1*) | 1# | CCGGGCTGAGAACTTAACTGCTGAACTCGAGTTCAGCAGTTAAGTTCTCAGCTTTTTG |
| | | AATTCAAAAAGCTGAGAACTTAACTGCTGAACTCGAGTTCAGCAGTTAAGTTCTCAGC |
| | 2# | CCGGGTCCTATTCCATGGTTCATTGCTCGAGCAATGAACCATGGAATAGGACTTTTTG |
| | | AATTCAAAAAGTCCTATTCCATGGTTCATTGCTCGAGCAATGAACCATGGAATAGGAC |
| *Glut3* (*Slc2a3*) | 1# | CCGGGCCATGAGCTTTGTCTGTATTCTCGAGAATACAGACAAAGCTCATGGCTTTTTG |
| | | AATTCAAAAAGCCATGAGCTTTGTCTGTATTCTCGAGAATACAGACAAAGCTCATGGC |
| | 2# | CCGGCTGTTACTAAAGGATGACTATCTCGAGATAGTCATCCTTTAGTAACAGTTTTTG |
| | | AATTCAAAAACTGTTACTAAAGGATGACTATCTCGAGATAGTCATCCTTTAGTAACAG |

**Supplementary Table 3: Primers for RACE used in this study.**

| Name | Description | Sequence (5' →3' ) |
|---|---|---|
| Universal Adapter | universal primer for 5' & 3' RACE PCR | AGCAGTGGTATCAACGCAGAGTAC |
| 3RACE-F1 | first round for 3' RACE | GAATAACAGTAGTTGTCTCCAGGC |
| 3RACE-F2 | second round for 3' RACE | GTCTCCAGGCTCCCAGTTTGTATTG |
| 5RACE-R1 | first round for 5' RACE | CCACCGAGTTCTTCAGCTGC |
| 5RACE-R2 | second round for 5'RACE | GCGGTGCGCGCTGCGTTTCGCC |
| Full length-F | forward primer | TCCACACCGGGGAACACTGAACCA |
| Full length-R | reverse primer | ACAAAACATGGATAAATTAAAAACTTC |

**Supplementary Table 4: sgRNA oligonucleotides used in this study.**

| Target gene | Description | sgRNA sequence (5'→3') |
|---|---|---|
| *Gm11549* | promoter KO | GGCCCTCAGAAAGTGGGGCG |
| | exon1 KO | GGAGAGCTAACTTCAGCGGG |
| | exon2 KO | GGTCCCGATGACATAGATCT |
| | exon3 KO-1 | GGGGACCAGGACTACGTCAC |
| | exon3 KO-2 | GTGACTAGTAGTTGCCGTAT |
| | dCas9-VP64-1 (CRISPRa) | GTGAACTACCGACGCATCCA |
| | dCas9-VP64-2 (CRISPRa) | GAAGGTGCGGTCTGAAGGGT |
| | dCas9-KRAB-1 (CRISPRi) | GGAGGCCGGGCGGACCACAG |
| | dCas9-KRAB-2 (CRISPRi) | GGCTCCGCACGCTCGCCCCC |
| | NEMEP frameshift mutant (*Nemep* KO) | GCAGAAAGAAGAAGAGCGCT |
| | 3xFLAG KI | GAGCTGTGTGACCCAGCTGGC |

**Supplementary Table 5: Genotyping PCR primers used in this study.**

| Name | Description | Sequence (5'→3') |
|---|---|---|
| PF | promoter KO | TTCAGGTGCCTAGGGAGTGG |
| PR1 | | GGAGAGACAACAGGCATTCTTCA |
| PR2 | | GCTCGTTATTGAAACCCCACG |
| K1F | exon1 KO | GAGCATCTCCCAACCCTTCA |
| K1R | | GTCCAGGACTAGACTCCTGT |
| K2F | exon2 KO | GTGGTGCCCAAGAGAAACGA |
| K2R | | GCAGTTTGTCCTGTCCTGAGA |
| K3F | exon3 KO | GATTCTTATGTGATCTCCGTGGCT |
| K3R | | AAAAGTTAAAACTATACTTCTAGTATGG |
| MF | NEMEP frameshift mutant (*Nemep* KO) | CACTTCACAGCTAGGAGCATCTC |
| MR | | AGCGACTCATTCTTCTACTCGC |
| KIF | 3xFLAG KI | ACACAGAAGATCTGCGGCG |
| KIR | | CAGTTCTCTGTGTTATCAGCCT |

**Supplementary Table 6: qPCR primers used in this study.**

| Gene | Forward sequence (5' →3') | Reverse sequence (5' →3') |
|---|---|---|
| *β-actin* | GTGACGTTGACATCCGTAAAGA | GCCGGACTCATCGTACTCC |
| *Gapdh* | CTCCACTCACGGCAAATTCA | CGCTCCTGGAAGATGGTGAT |
| *Foxh1* | GACCTGGCCTCAACTTACACT | TGTCATGCCGTAGGTATCTCTTC |
| *Gsc* | TTGCACAGACAGTCGATGCTACT | TCGTTGCTTTCTCGACCCC |
| *Mixl1* | CGGTTCTGGATCATCTCTCACA | TACCGAGAACAAGCCAGCAGT |
| *T/Brachyury* | TCCTCCATGTGCTGAGACTTGT | CCAAGAGCCTGCCACTTTG |
| *Fgf5* | AAGTAGCGCGACGTTTTCTTC | CTGGAAACTGCTATGTTCCGAG |
| *Foxa2* | CCCTACGCCAACATGAACTCG | GTTCTGCCGGTAGAAAGGGA |
| *Eomes* | ACCGGCACCAAACTGAGA | AAGCTCAAGAAAGGAAACATGC |
| *Sox17* | GCCGAGCCAAAGCGG | GTCAACGCCTTCCAAGACTTG |
| *Nkx2-5* | GACAAAGCCGAGACGGATGG | CTGTCGCTTGCACTTGTAGC |
| *U1* | CTGGCAGGGGAGATACCATG | AGTCGAGTTTCCCGCATTTG |
| *H19* | GAACAGAAGCATTCTAGGCTGG | TTCTAAGTGAATTACGGTGGGTG |
| *Sox1* | ATACCGCAATCCCCTCTCAG | ACAACATCCGACTCCTCTTCC |
| *Nestin* | CAGGATTGGGAGGAGGGCAGAG | GGAGGCAGGAGACTTCAGGTAG |
| *Pax6* | GTTCCCTGTCCTGTGGACTC | ACCGCCCTTGGTTAAAGTCT |
| *Tubb3* | GGCAACTATGTAGGGGACTCAG | CCTGGGCACATACTTGTGAG |
| *Lefty1* | TGTGTGTGCTCTTTGCTTCC | GGGGATTCTGTCCTTGGTTT |
| *Lefty2* | GGAACAGGTCCTGAGCAGTC | TCGAAAATTCTGGCTGAACC |
| *Glut1(Slc2a1)* | CAGTTCGGCTATAACACTGGTG | GCCCCCGACAGAGAAGATG |
| *Glut3(Slc2a3)* | ATGGGGACAACGAAGGTGAC | GTCTCAGGTGCATTGATGACTC |
| *Gm11549* | CTCCTATCACAGCCTAGGAGC | GGACTCAGACAGGCACATTC |

**Supplementary Data 1: Mass spectrometry analysis of co-purified proteins of GFP-EV and GFP-NEMEP.**

See Supplementary Data 1. xls.
