# Supplementary material for: A Nodal enhanced micropeptide NEMEP regulates glucose uptake during mesendoderm differentiation of embryonic stem cells
Source: Nat Commun. 2022 Jul 9;13:3984. doi: 10.1038/s41467-022-31762-x (PMC9271079; doi:10.1038/s41467-022-31762-x)
Supplement: Supplementary file 3 — Description of Additional Supplementary Files [file 41467_2022_31762_MOESM3_ESM.pdf]

Description of Additional Supplementary Files

Supplementary Data 1: Mass spectrometry analysis of co-purified proteins of GFP-EV and GFP-NEMEP.
