# Supplementary material for: A Nodal enhanced micropeptide NEMEP regulates glucose uptake during mesendoderm differentiation of embryonic stem cells
Source: Nat Commun. 2022 Jul 9;13:3984. doi: 10.1038/s41467-022-31762-x (PMC9271079; doi:10.1038/s41467-022-31762-x)
Supplement: Supplementary file 5 — Reporting Summary [file 41467_2022_31762_MOESM5_ESM.pdf]

nature portfolio

Corresponding author(s): Qiaoran Xi

Last updated by author(s): Jun 16, 2022

Reporting Summary

Nature Portfolio wishes to improve the reproducibility of the work that we publish. This form provides structure for consistency and transparency in reporting. For further information on Nature Portfolio policies, see our Editorial Policies and the Editorial Policy Checklist.

Statistics

For all statistical analyses, confirm that the following items are present in the figure legend, table legend, main text, or Methods section.

n/a	Confirmed	
☐	☒	The exact sample size (*n*) for each experimental group/condition, given as a discrete number and unit of measurement
☐	☒	A statement on whether measurements were taken from distinct samples or whether the same sample was measured repeatedly
☐	☒	The statistical test(s) used AND whether they are one- or two-sided<br>*Only common tests should be described solely by name; describe more complex techniques in the Methods section.*
☒	☐	A description of all covariates tested
☐	☒	A description of any assumptions or corrections, such as tests of normality and adjustment for multiple comparisons
☐	☒	A full description of the statistical parameters including central tendency (e.g. means) or other basic estimates (e.g. regression coefficient) AND variation (e.g. standard deviation) or associated estimates of uncertainty (e.g. confidence intervals)
☐	☒	For null hypothesis testing, the test statistic (e.g. *F*, *t*, *r*) with confidence intervals, effect sizes, degrees of freedom and *P* value noted<br>*Give P values as exact values whenever suitable.*
☒	☐	For Bayesian analysis, information on the choice of priors and Markov chain Monte Carlo settings
☒	☐	For hierarchical and complex designs, identification of the appropriate level for tests and full reporting of outcomes
☒	☐	Estimates of effect sizes (e.g. Cohen's *d*, Pearson's *r*), indicating how they were calculated

Our web collection on statistics for biologists contains articles on many of the points above.

Software and code

Policy information about availability of computer code

Data collection	NIKON A1R HD25; QuantStudio 7 Flex; BD Biosciences; Seahorse XFe96 analyzer
Data analysis	GraphPad Prism V9 ; Image J V1.50e; FlowJo v7.6 ; IGV_2.8.13; HiSeq Control (v3.4.0) Software;<br>NIS-Elements Viewer 4.11.0;TMHMM Server v2.0 ;Cufflinks v2.2.1 ;TopHat 2.0.6; RStudio (V1.2.1335);DeepTools(v 3.4.3) ;MACS2(v 2.1.4);TrimGalore(v 0.6.1);Bowtie2(v 2.3.3)

For manuscripts utilizing custom algorithms or software that are central to the research but not yet described in published literature, software must be made available to editors and reviewers. We strongly encourage code deposition in a community repository (e.g. GitHub). See the Nature Portfolio guidelines for submitting code & software for further information.

Data

Policy information about availability of data

All manuscripts must include a data availability statement. This statement should provide the following information, where applicable:

- Accession codes, unique identifiers, or web links for publicly available datasets
- A description of any restrictions on data availability
- For clinical datasets or third party data, please ensure that the statement adheres to our policy

The data discussed in this work have been deposited in NCBI's Gene Expression Omnibus and are accessible through GEO Series accession number GEO: GSE157073, GSE115169, GSE70486, GSE125116.
Source data are provided with this paper.

Field-specific reporting

Please select the one below that is the best fit for your research. If you are not sure, read the appropriate sections before making your selection.

☒ Life sciences ☐ Behavioural & social sciences ☐ Ecological, evolutionary & environmental sciences

For a reference copy of the document with all sections, see nature.com/documents/nr-reporting-summary-flat.pdf

Life sciences study design

All studies must disclose on these points even when the disclosure is negative.

Sample size	No specific methods were used for sample size determination. No statistical methods were used to predetermine sample size. Sample size are based on lots of previous publications and our previous experience, which is the most optimal to generate statistically significant results. All experiments were carried out at least three times. For each experiment, three biologically independent samples unless otherwise stated.Sample size for all the presented experiments was determined to be at least 3 to provide sufficient data for downstream analysis.
Data exclusions	No data were excluded
Replication	For each experiments the number of biological independent sample is reported in the figure legend. In vitro studies are represented at least 3 independent reproducible studies.
Randomization	All samples and animals were analysed and allocated randomly.
Blinding	The investigators were not blinded to allocation during experiments. Blinding was not possible as the same investigator performed genotyping and analysis.

Reporting for specific materials, systems and methods

We require information from authors about some types of materials, experimental systems and methods used in many studies. Here, indicate whether each material, system or method listed is relevant to your study. If you are not sure if a list item applies to your research, read the appropriate section before selecting a response.

Materials & experimental systems

n/a	Involved in the study	
☐	☒	Antibodies
☐	☒	Eukaryotic cell lines
☒	☐	Palaeontology and archaeology
☐	☒	Animals and other organisms
☒	☐	Human research participants
☒	☐	Clinical data
☒	☐	Dual use research of concern

Methods

n/a	Involved in the study	
☒	☐	ChIP-seq
☐	☒	Flow cytometry
☒	☐	MRI-based neuroimaging

Antibodies

Antibodies used	Mouse monoclonal anti FLAG® M2 antibody, Sigma-Aldrich Cat# F3165, clone:M2,1:3000;RRID:AB_259529<br>Mouse monoclonal anti GAPDH, ZSGB-BIO Cat# TA-08,clone:OTI2D9,1:5000; RRID:AB_2747414<br>Mouse monoclonal anti GFP, EASYBIO Cat# BE2001,clone:N/A, 1:2000; RRID:AB_2864742<br>Mouse monoclonal anti Histone H3, Abcam Cat# ab10799,clone:10799,1:10000; RRID:AB_470239<br>Mouse monoclonal anti SMAD2/3, CST Cat# 8685,clone:D7G7;1:1000 RRID:AB_2864743<br>Mouse monoclonal anti SMAD4, Santa Cruz Cat# sc-7966,clone:B-8,1:1000; RRID:AB_627905<br>Rabbit monoclonal anti CDH1, CST Cat# 3195,clone:24E10,1:1000; RRID:AB_2291471<br>Rabbit monoclonal anti FOXA2, CST Cat# 8186,clone:D56D6,1:200; RRID:AB_2864746<br>Rabbit monoclonal anti GLUT1,Beyotime Cat# AF1015,clone:N/A, 1:1000; RRID:AB_2864747<br>Rabbit monoclonal anti GLUT3, Abclonal Cat# A4137,clone:N/A, 1:1000; RRID:AB_2863194<br>Rabbit monoclonal anti HA Millipore, Cat# 04-902,clone:DW2, 1:2000; RRID:AB_1977526<br>Rabbit monoclonal anti Phospho-SMAD2/3, CST Cat# 8828,clone:D27F4,1:1000; RRID:AB_2864749<br>Rabbit monoclonal anti SMAD2,CST Cat# 5339, clone:D43B4,1:3000; RRID:AB_2864750<br>Rabbit monoclonal anti T/Brachyury, Abcam Cat# 209665, clone:EPR18113,1:200; RRID:AB_2864751<br>Rabbit polyclonal anti TRIM33, Bethyl Laboratories Cat# A301-060A; 1:1000,RRID:AB_2208024<br>IF488-Goat Anti-Rabbit IgG(H+L),huaxingbio,1:1000<br>Dylight 488-Donkey Anti-Goat IgG(H+L),huaxingbio,1:1000<br>DyLight594-Goat Anti-Mouse IgG (H+L),huaxingbio,1:1000
Validation	All antibodies were used according to the manufacturers' recommendations. All the companies provide quality certificates for the antibodies used in the study. Each antibody was first tested with different dilutions, and optimal dilution ratios used in the study are stated in the methods section.<br>Mouse monoclonal anti FLAG® M2 antibody, Sigma-Aldrich Cat# F3165; RRID:AB_259529,https://www.sigmaaldrich.com/US/en/product/sigma/f3165<br>Mouse monoclonal anti GAPDH, ZSGB-BIO Cat# TA-08; RRID:AB_2747414,http://www.zsbio.com/product/TA-08<br>Mouse monoclonal anti GFP, EASYBIO Cat# BE2001; RRID:AB_2864742,http://www.bioeasytech.com/product/2392.html?goods_id=4377<br>Mouse monoclonal anti Histone H3, Abcam Cat# ab10799; RRID:AB_470239,https://www.abcam.com/Histone-H3-antibody-mAbcam-10799-ChIP-Grade-ab10799.html<br>Mouse monoclonal anti SMAD2/3, CST Cat# 8685; RRID:AB_2864743,https://www.cellsignal.com/products/primary-antibodies/smad2-3-d7g7-xp-rabbit-mab/8685<br>Mouse monoclonal anti SMAD4, Santa Cruz Cat# sc-7966; RRID:AB_627905,https://datasheets.scbt.com/sc-7966.pdf<br>Rabbit monoclonal anti CDH1,CST Cat# 3195; RRID:AB_2291471,https://www.cellsignal.com/products/primary-antibodies/e-cadherin-24e10-rabbit-mab/3195<br>Rabbit monoclonal anti FOXA2, CST Cat# 8186; RRID:AB_2864746,https://www.cellsignal.com/products/primary-antibodies/foxa2-hnf3b-d56d6-xp-rabbit-mab/8186?_=1652559350699&Ntt=8186&tahead=true<br>Rabbit monoclonal anti GLUT1, Beyotime Cat# AF1015; RRID:AB_2864747,https://m.beyotime.com/Manual/AF1015%20Glucose%20Transporter%20GLUT1%20Rabbit%20Monoclonal%20Antibody.pdf<br>Rabbit monoclonal anti GLUT3, Abclonal Cat# A4137; RRID:AB_2863194, https://abclonal.com/Datasheet/Antibodies/A4137.pdf<br>Rabbit monoclonal anti HA Millipore, Cat# 04-902; RRID:AB_1977526, https://www.emdmillipore.com/US/en/product/Anti-HA-Tag-Antibody-clone-DW2-rabbit-monoclonal,MM_NF-04-902?ReferrerURL=https%3A%2F%2Fwww.bing.com%2F&bd=1<br>Rabbit monoclonal anti Phospho-SMAD2/3, CST Cat# 8828; RRID:AB_2864749, https://www.cellsignal.com/products/primary-antibodies/phospho-smad2-ser465-467-smad3-ser423-425-d27f4-rabbit-mab/8828<br>Rabbit monoclonal anti SMAD2,CST Cat# 5339; RRID:AB_2864750,https://www.cellsignal.com/products/primary-antibodies/smad2-d43b4-xp-rabbit-mab/5339<br>Rabbit monoclonal anti T/Brachyury, Abcam Cat# 209665; RRID:AB_2864751,https://www.abcam.com/brachyury--bry-antibody-epr18113-ab209665.html?productWallTab=ShowAll<br>Rabbit polyclonal anti TRIM33, Bethyl Laboratories Cat# A301-060A; RRID:AB_2208024, https://www.thermofisher.com/antibody/product/TRIM33-TIF1gamma-Antibody-Polyclonal/A301-060A<br>IF488-Goat Anti-Rabbit IgG(H+L),huaxingbio,http://www.huaxingbio.com/pd.jsp?id=176&nSL=%5B5%2C6%2C7%5D#skeyword=488-Goat+Anti-Rabbit+IgG&_pp=0_35<br>Dylight 488-Donkey Anti-Goat IgG(H+L),huaxingbio,http://www.huaxingbio.com/pd.jsp?id=180&nSL=%5B5%2C6%2C7%5D#skeyword=DyLight+488-Donkey+Anti-Goat+IgG&_pp=0_35<br>DyLight594-Goat Anti-Mouse IgG (H+L),huaxingbio,http://www.huaxingbio.com/pd.jsp?id=185&nSL=%5B5%2C6%2C7%5D#skeyword=DyLight594-Goat+Anti-Mouse+IgG&_pp=0_35

Eukaryotic cell lines

Policy information about cell lines

Cell line source(s)	Mouse ES cell lines E14Tg2a.IV ,HEK293T and HepG2 were purchased from the American Type Culture Collection (ATCC; https://www.atcc.org:443/)
Authentication	All the cells were authenticated using short-tandem repeat (STR) profiling by the provider ATCC.
Mycoplasma contamination	Cells were routinely tested for mycoplasma, and we confirm that every cell used in this study was found negative.

Commonly misidentified lines (See ICLAC register)	No commonly misidentified cell lines were used.

Animals and other organisms

Policy information about studies involving animals; ARRIVE guidelines recommended for reporting animal research

Laboratory animals	C57BL/6J mouse strains were obtained from Laboratory Animal Research Center in Tsinghua University. Female mice were used between ages 6-8 weeks of age. Mouse strains were housed at 20-22 degree with 12h:12h light:dark cycles at 50-60% humidity. The animal experiments that were conducted as part of this research were completed in accordance with the guidelines provided by the Tsinghua University Animal Care and Use Committee and were in compliance with the relevant ethical regulations regarding animal research.
Wild animals	The study did not involve wild animals.
Field-collected samples	The study did not involve samples collected in the field.
Ethics oversight	All animal procedures were approved by Institutional Animal Care and Use Committee (IACUC) of Tsinghua University.(IACUC:16-XQR2)

Note that full information on the approval of the study protocol must also be provided in the manuscript.

Flow Cytometry

Plots

Confirm that:

- [x] The axis labels state the marker and fluorochrome used (e.g. CD4-FITC).
- [x] The axis scales are clearly visible. Include numbers along axes only for bottom left plot of group (a 'group' is an analysis of identical markers).
- [x] All plots are contour plots with outliers or pseudocolor plots.
- [x] A numerical value for number of cells or percentage (with statistics) is provided.

Methodology

Sample preparation	For flow based sorting or the GFP positive cells,mESCs were digested with 0.25% Trypsin-EDTA and resuspended in 0.2 mL PBS. For flow studies of BiFC assay,the methods are provided in the Methods section.
Instrument	BD Calibur flow cytometer,BD FACS AriaII
Software	Data were collected and analyzed using FlowJo software.
Cell population abundance	For flow based sorting, the GFP positive cells(25%) were sorted out by FACS.
Gating strategy	For flow based sorting of the GFP positive cells, parental cells without plasmid transfection were used to define negative cell populations and set gates for analysis. For flow studies of BiFC, we use same cells without plasmid transfection for defining negative cell populations and set gates for analysis

- [x] Tick this box to confirm that a figure exemplifying the gating strategy is provided in the Supplementary Information.
